# Human pegivirus 1 in healthy blood donors and in acute febrile patients from Mato Grosso, Midwestern Brazil

**DOI:** 10.1007/s00705-026-06720-3

**Published:** 2026-08-28

**Authors:** Eduarda Pavan, Millena Moreira Carrasco, Janeth Aracely Ramirez Pavón, Leonardo Marin, Márcio Roberto Teixeira Nunes, Marcelo Adriano Mendes dos Santos, Renata Dezengrini

**Affiliations:** 1 Programa de Pós-graduação em Ciências da Saúde, Faculdade de Medicina, Universidade Federal de Mato Grosso, Cuiabá, MT Brazil; 2 Setor de Hemovigilância, MT-Hemocentro, Secretaria Estadual de Saúde, Cuiabá, Mato Grosso Brazil; 3 Laboratório de Tecnologia Biomolecular, Centro de Ciências Biológicas, Universidade Federal do Pará, Belém, PA Brazil; 4 Faculdade de Medicina, Universidade do Estado de Mato Grosso, Cáceres, MT Brazil; 5 Universidade Federal de Mato Grosso, Avenida Fernando Correa da Costa, 2367, Boa Esperança, Cuiabá - MT, Cuiabá, 78060-900 Brazil

## Abstract

The association between Human Pegivirus 1 (HPgV-1, *Pegivirus hominis*, family *Hepaciviridae*) with disease etiology remains unclear, yet this virus is widely distributed among people exposed to contaminated blood. This study aimed to identify the prevalence and characterize HPgV-1 genomes obtained from acute febrile patients (n = 92 from 2019) and from blood donors (n = 633 from 2022) sampled in Mato Grosso, Brazil. The serum samples were tested by RT-qPCR for a conserved 5’ UTR region of HPgV-1. Nucleic acid of 28 positive samples was converted to ds-cDNA, purified and sequenced with an Illumina NextSeq platform. The prevalence of HPgV-1 among acute febrile patients was 4.35% (4/92), who were mostly female (3/4; 75.00%) mean 28.86 (12–67) years-old; whereas among blood donors it was 3.79% (24/633), which were predominantly male (16/24; 66.67%), aged 30–45 years-old (13/24; 54.17%). Eight HPgV-1 genomic sequences (7,708-9,248) were recovered; two belong to subgenotype 2a and clustered with strains from United States, Brazil, France and Japan, while the remaining six genomes from subgenotype 2b grouped with strains from Pará, Brazil and abroad. HPgV-1 detection in acute febrile patients and in healthy blood donors highlights the necessity to investigate the epidemiological aspects of this infection, as well as the possible impact to public health in Midwestern Brazil.

**Supplementary Information:**

The online version contains supplementary material available at https://doi.org/10.1007/s00705-026-06720-3.

## Background

Formerly known as hepatitis G virus (HGV), Human Pegivirus type 1 (HPgV-1) is a *Hepaciviridae* family member of *Pegivirus* genus, firstly detected in patients with non-A, non-B, non-C viral hepatitis in 1995 [1, 2]

Pegiviruses have been grouped based on genetic divergence (0.31 and 0.31–0.36 aa p distance at NS3 and NS5, respectively) and host range. HPgV-1 currently presents seven genotypes, with predominance of genotypes 1 and 2 in America and Europe, whereas the remaining genotypes have been described in Asia and Africa [2, 3].

HPgV-1 is a blood-borne persistent virus with tropism for bone marrow cells such as lymphocytes [3–5]. Estimates indicate that 750 million people are viremic for HPgV-1, with a global prevalence of 3.1% in healthy general population, reaching 75.3% in individuals at high risk of contracting bloodborne infections in the developing world [4, 5]. In Brazil, HPgV-1 reports in blood donors indicate prevalence rates of up to 21.7% [6, 7].Although HPgV-1 is not associated with a specific disease etiology and is considered as a symbiont component of the human virome, recent studies conducted in Brazil have shown the presence of the virus genome in blood and cerebrospinal fluid of patients with fulminant hepatitis, encephalopathy and other nervous system infectious diseases of unknown etiology, and also related to an increased risk of lymphoma [8–10].

Using a metagenomic approach, our group has previously detected HPgV-1 genome in serum of patients with acute febrile disease [11]. This study was conducted to determine the prevalence of HPgV-1 and characterize its genomes by analyzing a representative sample of acute febrile patients suspected of having arbovirus infection in 2019 and of healthy blood donors sampled in 2022 in the state of Mato Grosso, Brazil.

## Materials and methods

### Study populations, sampling and ethical aspects

This observational cross-sectional study has been previously approved by the institutional human research ethics committee, under the registers 78203317.9.0000.8124 (approval 2.561.884 for acute febrile patients), and 54377421.8.0000.8124 (approval 5.291.983 for blood donors) and followed all regulations established by the National Health Council resolution CNS 460/2012, including the registration of free informed consent from participants.

Serum sample aliquots (*n* = 92) and epidemiological data collected from acute febrile patients suspected of arbovirus infection between January and December, 2019, sampled in basic health units of 22 municipalities of Mato Grosso and subjected to Dengue, Zika and Chikungunya virus diagnosis at LACEN-MT, were grouped into pools and subjected to Illumina NextSeq sequencing, as previously described [12]. Four pools from which HPgV-1 genomes were recovered via bioinformatic pipeline, had their serum samples tested individually for HPgV-1 in this study, except for four samples belonging to the same pool with insufficient volume (30–50 µL each), these serum samples were sub pooled and tested as one sample, resulting in 31 samples tested in two independent reactions.

Biological samples and epidemiological data from 633 blood donors over 18 years of age, obtained at the MT-Hemocentro after free informed and written consent from November 2021 to January 2023, as previously described [13], were tested individually in this study. Additionally, serum samples from these blood donors were tested for anti-HIV by chemiluminescence (Alinity m HIV-1 Assay, Abbott, USA) according to standard protocols and confirmed by NAT plus (Biomanguinhos/FIOCRUZ, Brazil).

### RT-qPCR for HPgV-1 detection

Each serum sample (0.4 mL) was subjected to nucleic acid extraction (MagMAX Viral/Pathogen Nucleic Acid Isolation Kit; Applied Biosystems/Thermo Fisher Scientific, Waltham, MA, USA) and immediately tested by one step RT-qPCR, which targeted a 5´UTR region of HPgV-1 (Forward 5’ - GGCCAAAAGGTGGTGGATGG − 3’; reverse 5’ - CTTAAGACCCACCTATAGTGGCTACC − 3’) as previously described [14–16]. Briefly, the reactions (30 µL) included 11 µL of nucleic acid, 7.5 µL of Master Mix (Allplex, Seegene), 0.2 µM of Taqman probe-ABI ([FAM] TGACCGGGATTTACGACCTACCAACCCT [TAMRA]-3’, 0.3 µM of each primer and 11.02 µL of ultrapure water and were reverse transcribed at 50 °C for 20 min, followed by initial denaturation at 95 °C for 2 min, and 40 cycles of 95 °C for 15 s and 58 °C for 1 min. All reactions included positive and negative serum controls, detected in a previous study [11]. Samples were considered positive when showing a CT < 40 in two independent tests.

### Library synthesis and Illumina NextSeq sequencing

Serum pools were processed and sequenced as previously described for both populations [12, 13]. The nucleic acid obtained from the serum of positive blood donors was converted to ds-cDNA and subjected to a random viral PCR, as previously described [14]. The resulting DNA product was quantified, subjected to QC sample evaluation on a 2100 Bioanalyzer (Agilent Technologies), used for library construction with TruSeq Nano DNA Kit (Illumina Inc, San Diego, CA, USA) and subjected to paired-end sequencing on the NextSeq 500/550 (300 cycles) platform.

### Bioinformatic pipeline

The genome assembly was performed using high-throughput Illumina sequencing data, following a comprehensive workflow for quality control, trimming, assembly, and manual curation. Initially, raw data was subjected to FastQC [15] to assess read quality and subsequently trimmed to remove adapters, short reads (< 75 nucleotides) and reads with nondetermined bases (> 10 N) using Trimmomatic [16]. De novo genome assembly with k-mer values of 22, 33, 55 and 77 was conducted using the SPAdes assembler v. 4.0.0 [17]. Post-assembly, taxonomic assignment and visualization were performed with Krona [18] and functional annotation against non-redundant protein data base with e-value cutoff of 0.0001 was facilitated by DIAMOND [19], a fast sequence aligner for protein-level homology detection. The final contigs were manually curated and validated using Geneious tool map to reference with minimum overlap value of 90% (v. 9, Biomatters, New Zealand) [20] to resolve ambiguities, eliminate misassemblies, and ensure overall assembly quality. The resulting contigs that share similarities with viral sequences were subjected to gene prediction using GeneMarkS [21] and to the NCBI Conserved Domains platform [22].

The HPgV-1 genomic sequences were individually analyzed with BlastX and with the plug-in ORFfinder, both implemented in Geneious v9, to predict the open reading frames (ORFs), 5′ and 3′-UTR. The alignment with the MAFFT v7.308 plug-in implemented on Geneious v9 [23] included 46 coding nucleotide sequences of all HPgV-1 genotypes available at GenBank (NCBI). Phylogeny was inferred using nucleotide coding region by maximum likelihood (ML), considering 1000 bootstraps replicates and GTR nucleotide substitution model at FastTree 2.1.11 [24] plugin implemented on Geneious [24]. The final phylogenetic trees were edited on FigTree v.1.4.4 [25] and on Inkscape (https://inkscape.org). HPgV-1 sequences retrieved in this study were deposited in GenBank database (accession numbers: PV107354-PV107361).

### Statistical analysis

Statistical analysis included a descriptive assessment of the study population and group comparisons using chi-square test in GraphPad Prism (v. 5, Dotmatics, Boston, MA. USA). Differences were considered significant when the p-value was < 0.05. Categorical variables were presented as absolute numbers and corresponding percentages. Occupations were classified according to the Brazilian Classification of Occupations (CBO) definitions.

## Results

### HPgV-1 prevalence in acute febrile patients

The acute febrile patients included in the study were mostly female aged 1–93 years, of brown ethnicity. HPgV-1 prevalence was 4.35% (4/92), three of these patients were female under 30 years-old and of brown ethnicity (Table 1). Statistical evaluation did not reveal a significant association between epidemiological variables.

**Table 1:** Epidemiological profile of acute febrile patients suspected of arbovirus infection tested for Human Pegivirus 1 in Mato Grosso State, West-Central Brazil

Variable/category	Total		HPgV (+)		HPgV (-)		*p* value
	*n*	%	*n*	%	*n*	%	
*Sex*							
Male	34	36.9	1	2.9	33	97.1	0.613
Female	58	63.1	3	5.2	55	94.8	
*Age (years)*							
< 30	58	63.1	2	3.5	56	96.5	0.314
30–45	19	20.7	1	5.3	18	94.7	
46–60	10	10.8	0	0	10	100.0	
> 60	5	5.4	1	20.0	4	80.0	
*Ethnicity*							
White	20	21.7	2	10.0	18	90.0	0.120
Brown	41	44.6	2	4.9	39	95.1	
Black	5	5.4	0	0	5	100.0	
Asian	1	1.1	0	0	1	100.0	
NI	25	27.2	0	0	25	100.0	

*NI* not Informed

HPgV-1 positive patients aged 12–67 years. The PV107355 pool was partially resolved, because of the lack of sufficient volume of four serum samples to repeat the nucleic acid extraction and the RT-qPCR tests individually. Therefore, these four serum samples were sub pooled and tested, whereas the remaining 30 samples were tested individually. Table 2 presents the clinical and demographic data of acute febrile patients positive for HPgV-1. Only one of these HPgV-1 positive samples was also diagnosed with Dengue virus infection at LACEN-MT.

**Table 2:** Clinical and epidemiological data of HPgV-1-positive acute febrile patients suspected of arbovirus infection in Mato Grosso, Central-Western Brazil

Patient	Age	Gender	Occupation	Race	City of residency	Symptoms and signals	RT-qPCR for arboviruses	RT-qPCR for HPgV-1 (CT)
PV107355 pool*	14–31	F (3), M (1)	NI (3) Student (1)	Brown (3) White (1)	Cuiabá	F (3), H (3), N (2), V (1), EP (1), BP (1), C (1)	1 Dengue vírus (1), negative (3)	39.7
*384*	67	M	Driver	White	Água Boa	F, M, H, JP, EP, P	Negative	37
*379*	32	F	Housewife	Brown	Cocalinho	F, M, H, C, BP, JP, EP	Negative	35.6
*353*	12	F	Student	White	Querência	F, M, H, JP, BP, P	Dengue virus	38

* Values in parenthesis refers to the number of patients included in the pool with that characteristic

*CT* Cycle threshold, *NI* Information unavailable, *F* fever, *H* headache, *N* nausea, *V* vomiting, *EP* eye pain, *M* myalgia, *C* conjunctivitis, *P* petechiae, *JP* joint pain, *BP* back pain

### HPgV-1 viremia in blood donors

The blood donors included in the study were mostly male, aged 17–71 years, self-reported to have brown ethnicity, with an overall HPgV-1 prevalence of 3.79% (24/633). The profile of these HPgV-1-positive donors indicates the majority were male with brown ethnicity and mean age of 38.54 (19–60) years (Table 3). Only one blood donor was co-infected with HIV.

**Table 3:** Epidemiological data of the blood donors tested for Human Pegivirus 1 in Mato Grosso, Brazil

Variable/category	Total		HPgV (+)		HPgV (-)		*p* value
	*n*	%	*n*	%	*n*	%	
Sex							
Male	345	54.5	16	4.6	329	95.4	0.297
Female	288	45.5	8	2.8	280	97.2	
*Age (years)*							
< 30	234	37.0	5	2.2	229	97.8	0.386
30–45	251	39.7	13	5.2	238	94.8	
46–60	128	20.2	6	4.7	122	95.3	
> 60	14	2.2	0	0	14	100	
NA	6	09	0	0	6	100	
*Race*							
White	158	25.0	7	4.4	151	95.6	0.960
Mixed race	238	37.6	10	4.2	228	95.8	
Black	84	13.3	2	2.4	82	97.6	
Indigenous	2	0.3	0	0	2	100	
Asian	4	0.6	0	0	4	100	
NI	147	23.2	5	3.4	142	96.6	
*Profession*							
No employment tie	128	20.3	3	2.3	125	97.7	0.736
Healthcare professional	35	5.5	1	2.9	34	97.1	
Higher education professional	177	28.0	9	5.1	168	94.9	
Secondary education professional	25	3.9	2	8.0	23	92.0	
Retail worker	75	11.9	3	4.0	72	96.0	
Military personnel	63	9.9	3	4.8	60	95.2	
Others	130	20.5	3	2.3	127	97.7	

### Molecular characterization of HPgV-1

In total, eight partial genome sequences were recovered from these populations, four from acute febrile patients (PV107354 - PV107357), and four from blood donors (PV107358 - PV107361). All sequences belong to genotype 2.

The best BLASTx results are shown in Supplementary Table A. All our sequences share a mean aa identity of 91.34% (82.52–99.56%) with the HPgV-1 genomes identified in France (PV107354-PV107356, PV107358, PV107360), in the United States (PV107357) and in Brazil (PV107359 and PV107361). The sequencing depth ranged from 9.8× to 90.4×, with an average genome coverage of 67.50%. Detailed sequencing quality metrics, including mapped reads, mean base quality, and BioProject information are provided in Supplementary Table B.

**Fig. 1:**
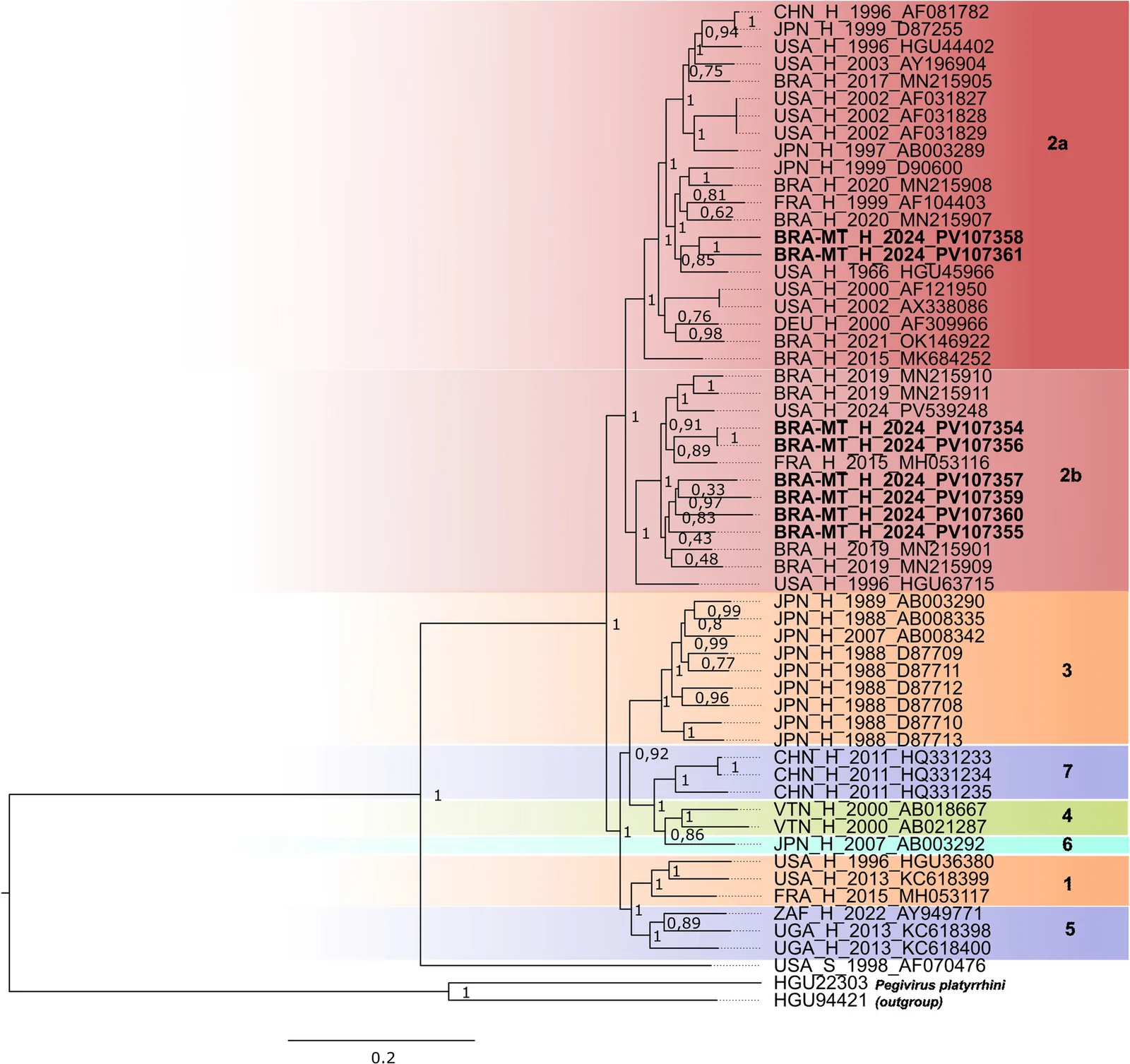
Maximum-likelihood phylogenetic analysis of nucleotide sequences from the coding region of the genomes recovered in this study (bold) compared with available sequences of Human Pegivirus type 1 (HPgV-1) genotypes 1–7 published in GenBank with accession numbers. The scale bar indicates the nucleotide substitutions per site. H: Human; S: simian; USA: United States of America; BRA: Brazil; CHN: China; JPN: Japan; DEU: Germany; FRA: France; UGA: Uganda; ZAF: South Africa; VTN: Vietnam

Maximum-likelihood phylogenetic analysis (Fig. 1) revealed that HPgV-1 sequences of the specie *Pegivirus hominis* separated into a well-supported monophyletic clade from the sequence of this same species identified in chimpanzee, AF070476. Thus, HPgV-1 can be separated into seven genotypes; all our eight sequences fit into genotype 2. Sequences PV107358 and PV107361, both retrieved from healthy blood donors, belong to subgenotype 2a, forming a monophyletic group (bootstrap = 1) with other sequences from the United States, Brazil, France and Japan, being closely related to sequence HGU45966 from the United States (bootstrap = 0.85). Subgenotype 2b separates into two clades where the other six sequences of this study stand in a monophyletic clade (bootstrap = 1) closely related to sequences from Pará, Brazil and other countries such as the United States and France. PV107354 and PV107356 form a smaller subclade (bootstrap = 1) between them.

Nucleotide and aminoacidic alignment matrix identities between all HPgV-1 sequences included in the phylogenetic analysis are described on Supplementary Table C. The nt and aa identities between our sequences and other HPgV-1 genomes used in our phylogenetic analysis are lower than 78% and 83%, respectively, because the polyprotein ORFs of our sequences are incomplete.

**Fig. 2:**
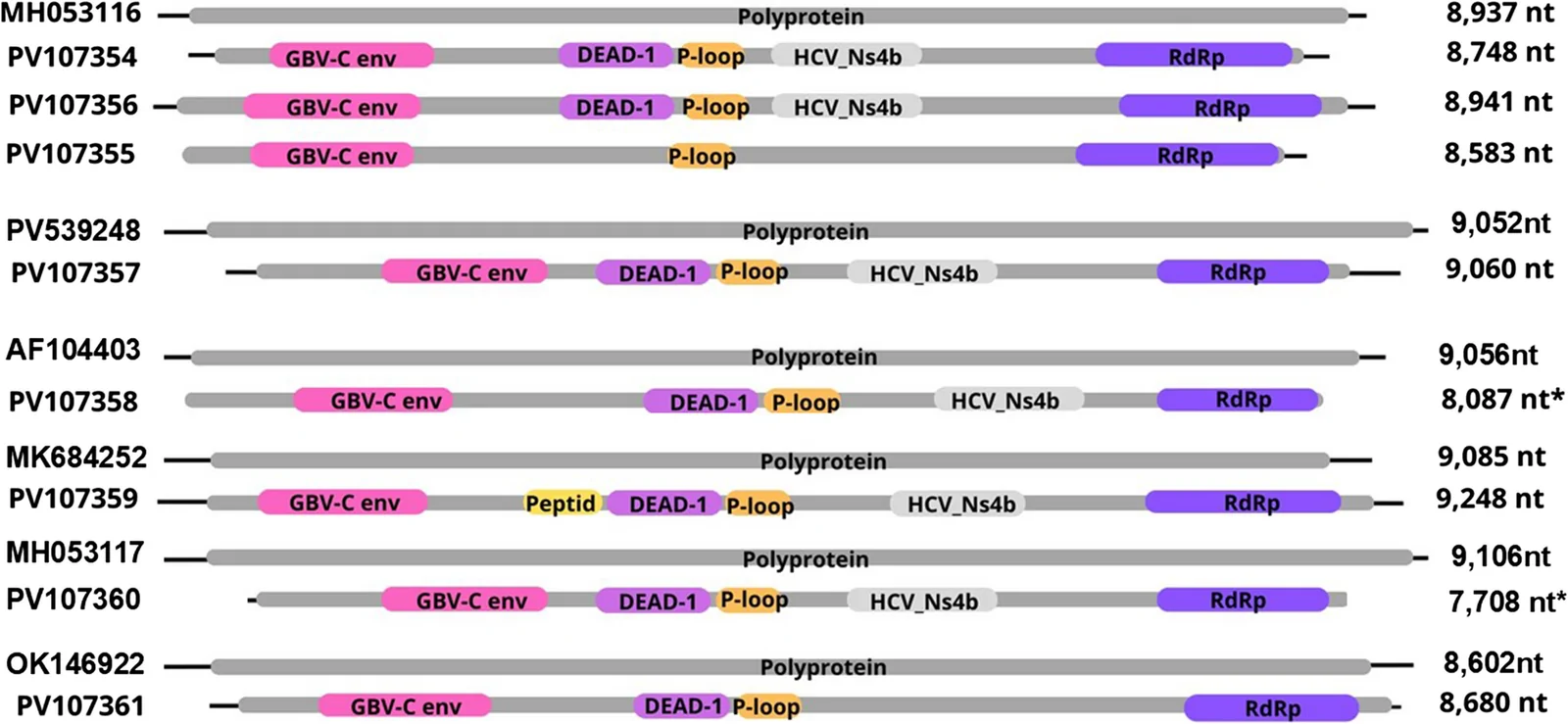
Genomic structure and characterization of the Human Pegivirus 1 (HPgV-1) sequences identified in this study. GBV-C: GB virus C genotype envelope; DEAD-1: DEAD-box helicase; HCV-NS4b: hepatitis C virus nonstructural protein NS4b; Peptid: hepatitis C virus NS3 protease; RdRp: RNA-dependent RNA polymerase. * Incomplete ORFs

Figure 2 presents a schematic representation of our HPgV-1 genomes compared with HPgV-1 sequences available in GenBank. These HPgV-1 sequences presented a mean length of 8,609 nt (7,708-9,248 nt). The conserved domains GBV-C env (cl15089), P-loop (cl38936) and RdRp (cl40470) were found in the eight sequences; the DEAD-box (cl28899) was found only in PV107354, PV107356, PV107357, PV107358, PV107360, PV107359 and PV107361. The HCV-NS4b (cl 03062) domain was found in the PV107354, PV107356, PV107357, PV107358 and PV107359 and the peptidase_S29 (cl03772) was detected only in the PV107359 sequence, because the polyprotein ORFs of our sequences are incomplete. The specific nucleotide positions of ORFs and domains and the gaps within coding regions are provided in Supplementary Table D.

## Discussion

Previously, in a metagenomic study involving acute febrile patients suspected of having arbovirus infection, sampled during a large Chikungunya virus ECSA outbreak in Mato Grosso in 2018, two HPgV-1 genomes belonging to genotypes 1 and 2a, were recovered from the serum of patients from Várzea Grande and Cuiabá, respectively [11].

When the genomic data obtained from acute febrile patients sampled during an ongoing Dengue virus 2 outbreak in 2019 were analyzed [12], four additional genomic sequences were found in samples from regionally distinct cities of Mato Grosso (Cuiabá, Água Boa, Querência and Cocalinho). Although we were not able to test individually four samples from these positive pools detected during the metagenomic approach, these findings with febrile patients prompted us to test a healthy population from the State, aiming to estimate HPgV-1 prevalence and its regional importance. Specifically, the main objective was to identify if the acute febrile patients would present a similar infection index, to understand wether co-infection with arboviruses as Dengue virus, could modulate HPgV-1 detection in these febrile patients.

HPgV-1 infection has been associated with a reduction in immune biomarkers activation during concomitant infectious diseases in humans [4, 26]. Other studies have shown a slightly higher prevalence of HPgV-1 in acute febrile patients than the data obtained in this study, reaching 8.00% [27]. HPgV-1 was detected in all blood donors who developed acute febrile symptoms immediately after donation [28]. The ambiguity of the clinical significance of HPgV-1 infection reinforces the need for further studies, especially to assess its relevance when associated with typical symptoms of arbovirus infections, such as fever, myalgia, headache, and petechiae.

HPgV-1 prevalence varies significantly according to exposure risk and economic factors [29]. Among blood donors from developed countries, HPgV-1 prevalence varies between 0.8% and 46.6%, whereas in South America the pooled prevalence is 9.10% [4–7].

The detected prevalence of HPgV-1 RNA among blood donors in this study was 3.79%, approximately 2.9 times lower than the average rates reported in hemotherapy centers from other Brazilian States, which range from 9.50% to 21.70% in the North and South regions, respectively [9, 10]. HPgV-1 viremia in blood donors has been related to low education and economic status, to countries in development [30–31], and risk factors such as mean age of 40 years, male sex and brown ethnicity in Brazil, consistent with the epidemiological profile of donors reported in this study [5, 32]. Our data may reflect regional differences, in addition to different selection criteria, testing protocols between our study and other studies developed in Brazil.

Considered a non-pathogenic virus, HPgV-1 screening in blood banks is not routinely recommended by regulatory agencies such as the World Health Organization (WHO), the Food and Drug Administration (FDA), and the Brazilian Health Regulatory Agency (ANVISA). The high endemicity of HPgV-1 in healthy populations could potentially reduce the number of blood donations [7]. Although HPgV-1 was initially described as a posttransfusion viral hepatitis agent, its role in cases of this pathology attributed to unidentified etiological agents is still under investigation [3]. Recent studies have associated HPgV-1 with nonhepatic conditions as well [33].

The only blood donor included in this study with confirmed HIV serology, was also positive for HPgV-1. The prevalence of co-infection described in our study is approximately five times lower than that described in other hemotherapy centers in Brazil and around the world, where the observed rate is approximately 28.00% [32]. The relatively positive outcome of HPgV-1 and HIV coinfection have been associated with host immune response modulation through different pathways [34].

HPgV-1 is currently classified into seven genotypes and multiple subgenotypes, according to nucleotide sequence heterogeneity, which can be partial or complete [35]. The differences in HPgV-1 genotypes geographic distribution seem to be related to their origin, evolution, and transmission mechanisms [2–3]. Genotype 1 is divided into five subgenotypes and is distributed in North America and Africa [36]. Genotype 2 (subgenotypes 2a and 2b) is reported mainly in North and South America, Asia and Europe, and represents the only genotype detected in this study [37–39]. Genotype 3 has a relatively higher prevalence in countries from Asia and South America [34], and genotypes 4 and 5 are more common in the Philippines and other Southeast Asian countries, whereas genotypes 6 and 7 circulate in Asia [40].

The phylogenetic analysis revealed that all HPgV-1 sequences detected in this study formed clades with genotype 2 sequences along with subgenotypes 2a and 2b. The prevalence reported in our study is consistent with that reported in other studies carried out in different hemotherapy centers in Brazil, including the Midwest Region of the country [11, 41]. Most of our sequences formed clades or even shared a greater paired identity with sequences obtained from blood donors in Pará, a neighboring State of MT. This grouping suggests possible regional circulation of HPgV-1, since RNA viruses present local genetic diversifications related to the specific geographic distribution of genotypes [42].

Although most of our genomes represent partial genomic sequences, with gaps in the ORFs (67.5% coverage), it was possible to identify conserved domains such as the GBV-C envelope, DEAD-box, P-loop, RdRp and NS4b which are characteristic of pegiviruses in most of our sequences; these domains are involved in HPgV-1 genome organization and replication mechanism, and might be also involved in the stablishment of the persistent viremia [43].

Study limitations include that the two study populations were sampled during different time periods. Despite being considered a nonpathogenic virus, several studies have sought to elucidate the relationship and interaction of HPgV-1 viremia in healthy and unhealthy individuals. Although further evidence is needed before routine screening of HPgV-1 can be considered in hemovigilance strategies at blood banks, these findings reinforce the relevance of HPgV-1 studies in different populations.

## Conclusion

Our cross-sectional study described for the first time the prevalence of HPgV-1 in blood donors and patients with acute febrile illness in the state of Mato Grosso, Brazil, sharing one co-infection with HIV in a blood donor and another co-infections with Dengue virus in a febrile patient. Phylogenetic analysis revealed that all the genomic sequences of the study belong to genotype 2, with a relatively higher prevalence of subgenotype 2B, the most common in Brazil. Further studies are needed to determine the causal relationship of the virus, if any, with the pathophysiology of acute febrile illnesses and other concurrent infections as HIV, as well as the importance of HPgV-1 for surveillance at blood banks.

## Supplementary Information

Below is the link to the electronic supplementary material.

Supplementary Material 1 (XLSX 36.3 KB)

## Data Availability

The dataset(s) supporting the conclusions of this article are available upon reasonable request to correspondence author. Raw sequencing data were deposited in the genbank repository, as bioinformatic bioprojects: IDs PRJNA 909359, PRJNA 1216278 and PRJNA909359, available at https://www.ncbi.nlm.nih.gov/bioproject.
